# Complete Genome Sequencing, Molecular Epidemiological, and Pathogenicity Analysis of Pigeon Paramyxoviruses Type 1 Isolated in Guangxi, China during 2012–2018

**DOI:** 10.3390/v12040366

**Published:** 2020-03-26

**Authors:** Ying He, Bingxia Lu, Kiril M. Dimitrov, Jiaxing Liang, Zhongwei Chen, Wu Zhao, Yibin Qin, Qunpeng Duan, Yingning Zhou, Lei Liu, Bin Li, Lingtian Yu, Zhenhua Duan, Qi Liu

**Affiliations:** 1 College of Animal Science and Technology, Guangxi University, Nannnig 530004, China; yinghe2@gmail.com; 2 Guangxi Key Laboratory of Veterinary Biotechnology, Guangxi Veterinary Research Institute, Nanning 530001, China; lubingxia13@163.com (B.L.); liangjiaxing1964@163.com (J.L.); chen_zhong-wei@163.com (Z.C.); zhaowu168866@163.com (W.Z.); qinyibin5188@163.com (Y.Q.); 15107717676@163.com (Q.D.); zhouyingning1975@163.com (Y.Z.); libin2004a@126.com (B.L.); 3 Molecular and Virology Diagnostics Section, Texas A&M Veterinary Medical Diagnostic Laboratory, Amarillo, TX 79106, USA; 4 Department of Animal Science and Technology, Guangxi Agricultural Vocational College, Nanning 530007, China; gxullei@163.com (L.L.); ylt254523499@163.com (L.Y.); 5 Department of Testing & Quarantine, Animal Disease Control Center of Yulin, Yulin 537000, China; 15878546009@163.com

**Keywords:** pigeon paramyxovirus 1, next-generation sequencing, phylogenetic analysis, Newcastle disease

## Abstract

Newcastle disease is an important poultry disease that also affects Columbiform birds. The viruses adapted to pigeons and doves are referred to as pigeon paramyxoviruses 1 (PPMV-1). PPMV-1 are frequently isolated from pigeons worldwide and have the potential to cause disease in chickens. The complete genomes of 18 PPMV-1 isolated in China during 2012–2018 were sequenced by next-generation sequencing (NGS). Comprehensive phylogenetic analyses showed that five of the viruses belong to sub-genotype VI1.2.1.1.2.1 and 13 isolates belong to sub-genotype VI.2.1.1.2.2. The results demonstrate that these sub-genotypes have been predominant in China during the last decade. The viruses of these sub-genotypes have been independently maintained and continuously evolved for over 20 years, and differ significantly from those causing outbreaks worldwide during the 1980s to 2010s. The viral reservoir remains unknown and possibilities of the viruses being maintained in both pigeon farms and wild bird populations are viable. In vivo characterization of the isolates’ pathogenicity estimated mean death times between 62 and 114 h and intracerebral pathogenicity indices between 0.00 and 0.63. Cross-reactivity testing showed minor antigenic differences between the studied viruses and the genotype II LaSota vaccine. These data will facilitate PPMV-1 epidemiology studies, vaccine development, and control of Newcastle disease in pigeons and poultry.

## 1. Introduction

Newcastle disease virus (NDV), commonly named avian paramyxovirus 1, is member of the genus *Orthoavulavirus* of the family *Paramyxovorodae* [1]. The virus has significant genetic diversity and is one of the most economically important avian pathogens in the world. Virulent strains cause Newcastle disease (ND), which is devastating, with up to 100% mortality in naïve chickens [2]. There are at least 21 identified genotypes of NDV, separated into two classes—class I and class II [3]. Viruses of class II genotypes VI and XXI, antigenic variants of NDV, have been identified in many species [4], but predominantly affect birds of the family *Columbidae* (doves and pigeons), and are commonly referred to as pigeon paramyxoviruses 1 (PPMV-1) [5]. NDV is an enveloped virus with a single-stranded, negative-sense, non-segmented RNA that encodes for at least six proteins: nucleoprotein (NP), phosphoprotein (P), matrix (M), fusion (F), hemagglutinin-neuraminidase (HN), and large polymerase (L) (3′-NP-P-M-F-HN-L-5′) [6].

Newcastle disease viruses are categorized into three main pathological groups based on the clinical signs they cause in chickens: (i) lentogens are of low virulence or avirulent and cause no clinical disease to mild enteric, respiratory, or subclinical disease; (ii) mesogens cause disease and death primarily in chickens younger than eight weeks and produce mainly respiratory disease; and (iii) velogens induce severe systemic infections with high mortality rates [7,8]. Pigeons of all ages are susceptible to infection with NDV and the morbidity and mortality rates often exceed 50% [9]. At least five panzootics of ND have been described [10], and the third one, which is still ongoing, is in Columbiform birds. This panzootic in pigeons and doves emerged in the Middle East during the 1970s and quickly spread to many countries around the world [8,11].

PPMV-1 was introduced to Hong Kong in 1985, subsequently spread throughout China, and now poses a serious threat to the Chinese pigeon breeding industry and wild birds population [12]. The virus is now one of the most destructive pathogens for pigeons in the country, also affecting pigeon farms with increased frequency (Awu et al. 2015; Guo et al. 2013; Ren et al. 2016; Wei et al. 2018).

A commercial vaccine for the prevention of ND in pigeons is not available in China. Some pigeon farms have extensively been using chicken genotype II vaccines, such as LaSota and Clone-30, in attempts to protect pigeons from ND [13].However, these vaccines have not been fully efficient and the disease has broken in many pigeon farms in China. Previous studies have identified that the predominant viruses causing ND in pigeons in China between 1996 and 2015 were of sub/genotypes VIb (now named VI.1) and VII [14,15]. More recent pigeon-derived PPMV-1 were classified as members of sub-genotypes Vik and VIj [16], which are closely related to the viruses studies here.

While there are several studies of PPMV-1 in China, complete genome sequences are scarce and a contemporary classification of these viruses utilizing the newly accepted classification system is not available. The work described here was performed with the following aims: (i) to sequence the complete genomes of recent PPMV-1 isolated in China; (ii) to perform comprehensive phylogenetic analysis of the studied viruses; (iii) to study the molecular epidemiology of these viruses and their relationships to other PPMV-1; and (iv) to biologically characterize the pathogenicity and antigenicity of the studied PPMV-1. To this end, eighteen isolates were subjected to next-generation sequencing and the obtained genomes were molecularly and phylogenetically analyzed. The viruses were additionally characterized by establishing mean death times, intracerebral pathogenicity indices, and cross-reactivity to the most commonly used vaccine strain LaSota. The obtained data will facilitate future epidemiological studies of PPMV-1 and may be used to improve the prevention and control of ND in the pigeon industry.

## 2. Materials and Methods

### 2.1. Ethics Statement

This study was conducted in compliance with the “Guidelines for the Humanitarian Governance of Laboratory Animal Welfare” of the National Development and Reform Commission of the People’s Republic of China, and was approved by the Ethics Committee of Animal Welfare and Animal Experiments of the Guangxi University (Approval code: 2012005, Approval date: 6 January 2012). All procedures were conducted in accordance with the Animal Welfare Act and Guide for the Care and Use of Laboratory Animals (https://www.ncbi.nlm.nih.gov/books/NBK54050/).

### 2.2. Virus Isolation and Identification

Between 2012 and 2018, clinical samples were collected from pigeons with clinical signs of disease from farms in Guangxi Province, China. Among the clinical manifestations of the diseased birds were sagging wings, torticollis, and diarrhea, which are typical for ND in pigeons. Upon gross examination, hemorrhages and necrosis in the spleen and hemorrhages of the mucosa of glandular stomach were observed (Table 1). Tissue samples collected from diseased pigeons included brain, kidney, liver, and spleen. The tissues were weighed, homogenized, and diluted in sterile saline to a 10% (weight/volume) concentration. After ultrasonic treatment, the samples were centrifuged at 3000 rpm for 10 minutes. The supernatant was supplemented with penicillin and streptomycin (2000 IU/mL) and stored overnight at 4–8 °C before further use. Suspension supernatant (0.2 mL) from each sample was inoculated into five 9-to-11-day-old specific-pathogen-free (SPF) embryonating chicken eggs (ECE) using standard methods as described previously [17]. The allantoic fluids from inoculated ECEs were harvested and subjected to a hemagglutination assay (HA) using the microtiter method. Hemagglutinating agents were confirmed to be NDV by hemagglutination inhibition (HI) assay [18]. All allantoic fluids were shown to be negative for AIV H5, H7, and H9 through HI assay using positive serums (Merial-Vital, Beijing, China). All isolates were stored at −80 °C for further testing.

### 2.3. Determination of Minimal Lethal Dose, Mean Death Time, and Intracerebral Pathogenicity Index

Filtered allantoic fluids were serially diluted ten-fold from 10^−1^ to 10^−9^. One hundred microliters of every dilution were inoculated into five 9-to-11-day-old SPF ECE and incubated at 37 °C. Eggs were candled twice daily for viability for seven days and mortality times were recorded. The minimum lethal dose (MLD) was the highest dilution that caused death of all chicken embryos. Mean death time (MDT) was determined as the mean time in hours necessary for the death of all ECE that received MLD. MDT values between 60 and 90 h indicate moderate virulence, MDT values greater than 90 h indicate low virulence, and MDT values less than 60 h are indicative of virulent viruses. An intracerebral pathogenicity index (ICPI) assay in one-day-old chickens was also conducted on all viruses following established procedures [19].

### 2.4. Cross-Reactivity Testing

The LaSota vaccine and ten pigeon-derived PPMV-1 isolates (GXG2, GXG7, GXG16, GXG6/2015, GXG1, GXG3, GXG6/2013, GXG13, GXG24, and GXG25), representative for the studied groups of viruses, were selected for the cross-reactivity testing. Positive antisera were prepared as described previously [20]. Briefly, four-week-old SPF chickens were intramuscularly inoculated with inactivated virus (inoculation dose 10^6^ EID_50_ per chicken). After two weeks, a booster dose of each inactivated virus was administered to the respective chickens. Serum was collected from each bird 21 days after the booster, inactivated at 56 °C for 30 mins, and stored at −80 °C for further use. Negative sera were collected from un-inoculated SPF chickens. Cross-inhibition was evaluated using the collected positive polyclonal antisera, the LaSota vaccine, and the ten pigeon-derived PPMV-1 antigens following the standard HI procedure [19]. The titer was expressed as the highest dilution of antisera, which caused a complete inhibition of agglutination. The correlation coefficients of antigen homology were calculated according to the immunological method described by Archetti and Horsfall [21] using the following formulas:

$$R=\;r1\;\sqrt{r1\times \;r2}$$

$$r1=\frac{\mathrm{hemagglutination}\;\mathrm{inhibition}\;\mathrm{titer}\;\mathrm{of}\;\mathrm{serum}\;A\;\mathrm{against}\;\mathrm{antigen}\;B}{\mathrm{hemagglutination}\;\mathrm{inhibition}\;\mathrm{titer}\;\mathrm{of}\;\mathrm{serum}\;A\;\mathrm{against}\;\mathrm{antigen}\;A}$$

$$r2=\frac{\mathrm{hemagglutination}\;\mathrm{inhibition}\;\mathrm{titer}\;\mathrm{of}\;\mathrm{serum}\;B\;\mathrm{against}\;\mathrm{antigen}\;A}{\mathrm{hemagglutination}\;\mathrm{inhibition}\;\mathrm{titer}\;\mathrm{of}\;\mathrm{serum}\;B\;\mathrm{against}\;\mathrm{antigen}\;B}$$

The *R* value represents the difference in antigenicity between two viruses. Cross-reactivity *R* values higher than 0.80 prove antigenic identity; while those between 0.80 and 0.33 show antigenic relatedness with minor differences; and *R* values between 0.32 and 0.11 indicate loose relatedness, meaning major differences. Finally, *R* values below 0.11 indicate no relatedness at all, indicating different serotypes [22].

### 2.5. RNA Extraction, Next-Generation Sequencing (NGS) Library Preparation, Sequencing, and Genome Assembly

For the studied isolates, total RNA was isolated from the allantoic fluid using the QIAamp RNA viral mini kit (Qiagen, Hilden, North Rhine-Westphalia, Germany) and processed by next-generation sequencing using KAPA Stranded RNA-Seq kit (KAPA Biosystems, USA),as previously described [23,24]. All NGS libraries underwent equimolar dilution and were pooled. The library pool (600 μL) was loaded into the flow cell of the 300 cycle MiSeq Reagent Kit v2 (Illumina, San Diego, CA, USA) and pair-end sequencing (2 × 150 bp) was performed on the Illumina MiSeq instrument (Illumina, USA). After automated cluster generation in MiSeq, the sequencing reads were processed and all statistical data generated by the instrument were collected and summarized.

Data analysis and genome assembly were performed according to a previously described bioinformatics workflow [25] with some modifications. Host genome reads were removed using Burrows-Wheeler-Alignment Tool (BWA) (host sequence of chicken: GCF_000002315. 4_Gallus_gallus-5.0 (NCBI for GCF_000002315.5_grcg6a) and pigeon: GCF_000337935. 1 cliv_1.0 (https://www.ncbi.nlm.nih.gov/genome/10719)). Assembly of the raw reads and scaffolding were done using SOAP denovo. Mummer software v.3.0 (Stefan Kurtz, University of Hamburg, Hamburg, Germany) was used to align the assembled contigs to a reference sequence (KM374059.1) to further optimize the final assembly.

### 2.6. Collection of Sequences and Evolutionary and Phylogenetic Analysis

The class II complete F-gene dataset provided by Dimitrov et al., [3] and deposited in GitHub (https://github.com/NDVconsortium/NDV_Sequence_Datasets) was used (*n* = 1756 sequences). An additional search in GenBank (National Center for Biotechnology Information) [26] to check for recent submissions that are not included in the provided dataset was done (as of 5 February 2020). All collected sequences were aligned using Multiple Alignment with Fast Fourier Transformation (MAFFT v7.4.50) [27], as implemented in Geneious Prime (Biomatters Ltd., New Zealand).

The estimates of average evolutionary distances were inferred from the complete F gene sequences using MEGA6 [28]. Analyses were conducted using the maximum composite likelihood model [29]. The rate variation among sites was modelled with a gamma distribution (shape parameter = 1).

A maximum-likelihood tree was built based on general time-reversible (GTR) model [30] (goodness-of-fit measured by the corrected Akaike information criterion in MEGA6) using RaxML version 8.2.11 [31] with 1000 bootstrap replicates. A discrete Gamma distribution (Γ) was used to model evolutionary rate differences among sites and the rate variation model allowed for some sites to be evolutionarily invariable (I). The RaxML tree was constructed through the CIPRES Science Gateway [32]. The tree was inferred using the complete class II full-length F-gene alignment (*n* = 1787, including the original dataset of 1756 sequences, the sequences obtained here, and recent GenBank submissions). For imaging purposes, a smaller dataset (*n* = 110) including the sequences of the 18 viruses from this study, closely related PPMV-1, and representative viruses of genotype VI was parsed and used to build a smaller tree using the same method. In addition, closely related complete genome sequences were downloaded from GenBank and aligned as described above. The leader and tail sequences and intergenic regions were trimmed, and the coding sequences for all six genes were concatenated. A total of 108 concatenated complete genome coding sequences were used to infer a complete genome tree using the method used for the full fusion trees. All trees were visualized using FigTree v1.4.2 (http://tree.bio.ed.ac.uk/software/figtree). The Roman-Arabic numerals presented in the taxa names in the phylogenetic trees represent the respective genotype for each isolate, followed by the GenBank accession number, host name (if available), country of isolation, strain designation, and year of isolation. Recently described unified criteria [3] based on the phylogenetic topology and evolutionary distances between different taxonomic groups were used to determine genotypes and sub-genotypes. Amino acid alignments at the F protein cleavage site, functional domains, and neutralizing epitopes were compared to genotype II vaccine viruses LaSota, B1, and Clone 30 (GenBank accession numbers AY077761, AF309418, and Y18898, respectively) for substitutions using the MEGA6 software.

### 2.7. Antigenicity and Hydrophilicity Analysis of F and HN Proteins

Amino acid sequences of F and HN proteins in NDV isolates were deduced from the obtained nucleotide sequences. The Protean module of Laser gene under the Jameson–Wolf and Kyte Doolittle methods(DNASTAR, Madison, WI, USA) was used for antigenicity and hydrophilicity analyses of F and HN proteins, as described previously [18]. The sequences of three representative PPMV-1 (GXG2, GXG3, and GXG35) were compared to that of LaSota (GenBank accession number AY077761).

### 2.8. GenBank Accession Numbers

The complete genome sequences (*n* = 18) of PPMV-1 obtained in this study were submitted to GenBank and are available under the accession numbers from MK469964 to MK469974, MK749297 to MK749302, and MN477454.

## 3. Results

### 3.1. Virus Isolation and In Vivo Pathogenicity Characterization

A total of 18 PPMV-1 were isolated from the sampled pigeon farms between 2012 and 2018 (Table 1). The hemagglutination titers of the isolates ranged from log_2_3 to log_2_10. The mean death times of 11 isolates varied between 60 and 90 h. Such MDTs are specific for viruses that are mesogenic (of moderate virulence) for chickens. Seven isolates presented MDT above 90 h, which is typical for viruses that are lentogenic for chickens. Biological assessment of the pathogenicity of selected isolates was also performed by ICPI test in one-day-old SPF chickens. Thirteen viruses tested in the ICPI assay did not cause any clinical symptoms or mortality in one-day-old chickens and presented ICPI values of 0.00 (Table 1), which characterizes them as avirulent for chickens based on the World Organization for Animal Health (OIE) standards [19]. Five viruses had ICPI values between 0.17 and 0.63 (Table 1). Such ICPI values are specific for viruses that are of low virulence for chickens [17].

### 3.2. Whole Genome Sequencing and Phylogenetic Analyses

Next-generation sequencing of the 18 pigeon-origin viruses studied here yielded a total of 245,817,668 raw reads, of which 228,455,568 reads passed quality filtering. After removing the host reads and performing genome assembly, 60,883,067 reads were mapped as PPMV-1. Complete genomes were obtained for all studied viruses. The average depth was 222X with 90% of the assemblies having a mean depth above 100X. The full-length genome of all 18 viruses was 15,192 bp, following the “rule of six” and the standard NDV genome structure and gene order (5′-NP-P-M-F-HN-L-3′). Deduced amino acid analysis at the F protein cleavage site of all viruses predicted a motif with multiple basic amino acids (^113^RQKR↓F^117^), which is typical for viruses that are virulent for chickens.

To further characterize the studied PPMV-1, we performed distance analysis of these isolates. The sequences were used to determine the evolutionary distances among them and between them and closely related PPMV-1. Distance analyses revealed the separation of the 18 isolates into at least four groups, each of which consisted of closely related viruses. The first group (group 1) included five viruses isolated between 2012 and 2018 (isolate designations GXG2, GXG6/2015, GXG7, GXG16, and GXG44). The nucleotide identity within the group was high and varied between 98.5% and 99.8%. The remaining 13 viruses isolated between 2012 and 2017 (isolate designations GXG1, GXG3, GXG6/2013, GXG13, GXG20, GXG22, GXG24, GXG25, GXG28, GXG29, GXG31, GXG33, and GXG35) fell into three separate groups (group 2, 3, and 4)and seemed to be closely related with nucleotide distances between the three groups ranging between 0.9% and 2.0%. The viruses from group 1 were genetically distant from groups 2, 3, and 4 described above, with mean differences between the groups of 6.5%, 5.8%, and 6.0%, respectively. Analyses of the evolutionary distances of the isolates sequenced here showed that the nearest related viruses belong to class II genotype VI. The viruses from group 1 were closest to sub-genotype VI.2.1.1.2.1 with 96.3% nucleotide identity, and those from groups 2, 3, and 4 were closely related to sub-genotype VI.2.1.1.2.2 with 98.4% nucleotide identity.

**Figure 1:**
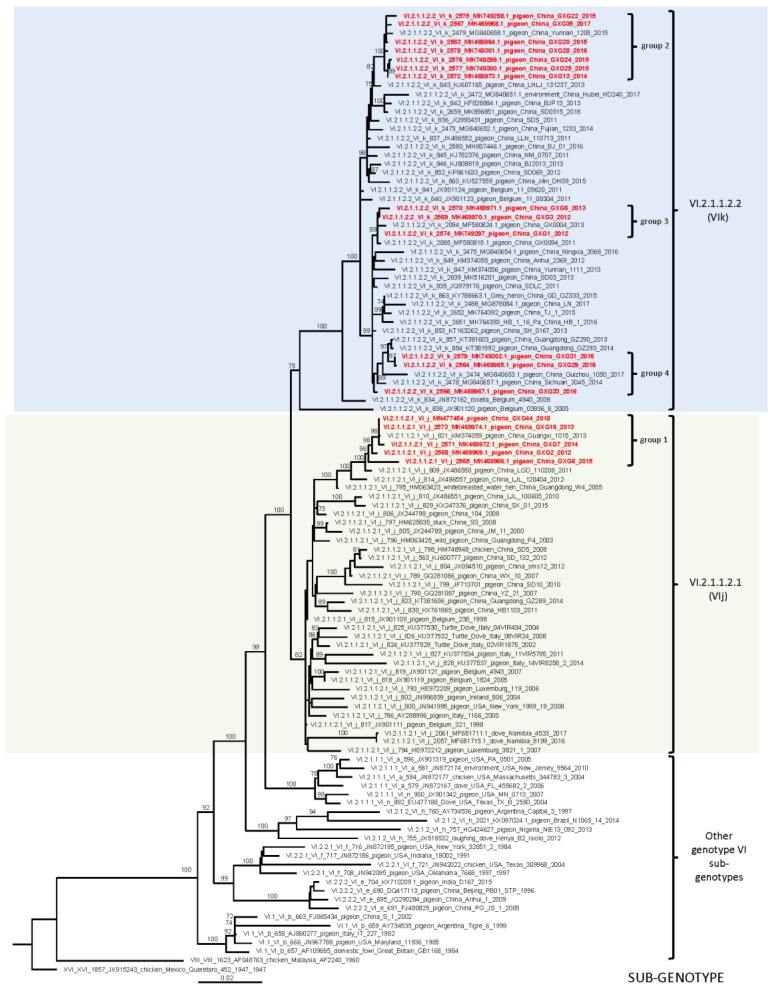
Phylogenetic analysis based on the full-length nucleotide sequence of the fusion gene of isolates representing Newcastle disease virus class II, sub-genotypes VI.2.1.1.2.1 and VI.2.1.1.2.2. The evolutionary history was inferred using the maximum likelihood method based on the general time reversible model with 1000 bootstrap replicates. The tree with the highest log likelihood (−12,395.64) is shown. A discrete gamma distribution was used to model evolutionary rate differences among sites. The analysis involved 110 nucleotide sequences with a total of 1662 positions in the final dataset. The viruses isolated in this study are designated in red bold font. The Roman numerals presented in the taxa names in the phylogenetic tree represent the respective sub/genotype for each isolate, followed by the GenBank identification number, host name, country of isolation, strain designation, and year of isolation (if available).

To study the evolutionary relationship between the isolated PPMV-1 studied here and viruses isolated in other geographical locations, a phylogenetic analysis was performed. Following recently published unified criteria for classification of NDV, a complete tree using all available full-length F-gene sequences of class II viruses (*n* = 1787) was built. This phylogenetic analysis confirmed the nucleotide distance results and classified all 18 isolates studied here as members of class II genotype VI (Supplemental Figure S1).The isolates from group 1 were identified as members of sub-genotype VI.2.1.1.2.1 (former VIj) and the remaining 13 isolates falling in three smaller groups belonged to sub-genotype VI.2.1.1.2.2 (former VIk). The full fusion tree results were confirmed by the complete genome phylogenetic inference (Supplemental Figure S2). For imaging purposes, a smaller full fusion tree, using 110 sequences (the 18 studied here with closely related viruses from sub-genotypes VI.2.1.1.2.1 and VI.2.1.1.2.2, and representative viruses from the remaining sub-genotypes of genotype VI), was built (Figure 1). Expectedly, the isolates studied here clustered together with the viruses to which they showed the highest nucleotide identity. The isolates from group 1 formed a monophyletic branch within sub-genotype VI.2.1.1.2.1 with viruses isolated in China from pigeons during 2001–2014 and a white-breasted water hen in 2005. The isolates from groups 2, 3, and 4 clustered into separate monophyletic branches within sub-genotype VI.2.1.1.2.2. Group 2 clustered with Chinese pigeon viruses from 2015, the viruses from group 3 formed a branch with Chinese pigeon viruses isolated in 2013, 2014, and 2017.Those from group 4 fell into a branch with Chinese pigeon viruses isolated during 2011–2013 (Figure 1, Supplemental Figure S1). Of note, viruses from these two sub-genotypes have been identified in other countries too. More than twenty isolations of viruses of sub-genotype VI.2.1.1.2.1 from Belgium, Italy, Luxemburg, USA, Ireland, and Namibia have been reported. The isolation of another seven viruses of sub-genotype VI.2.1.1.2.2 from Belgium, India, and Egypt has also been documented. The viruses of sub-genotype VI.2.1.1.2.1 and VI.2.1.1.2.2 have relatively high intra-populational diversity with 3.0% and 1.6% mean genetic distances. The addition of new sequences did not change the architecture of class II genotype VI NDV. All currently classified sub-genotypes maintained inter-populational evolutionary distance above 5% (Table 2).

### 3.3. Serum Cross-Reactivity, Antigenicity, and Hydrophilicity Testing

**Figure 2:**
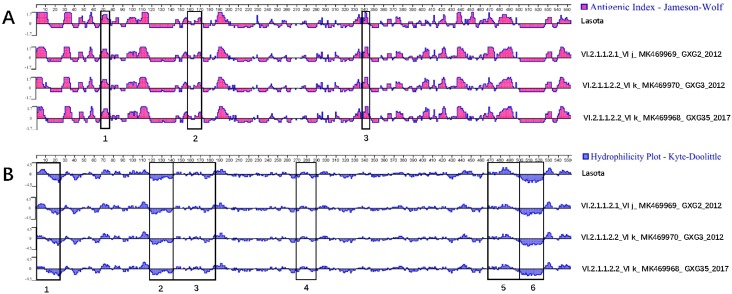
Comparison of the antigenicity and hydrophilicity of the F protein of selected PPMV-1 and LaSota vaccine. Protean module of Laser gene was used to analyze the antigenicity and hydrophilicity of fusion (F) protein of PPMV-1 isolates. GXG3 and GXG35 are representative isolates of sub-genotype VI.2.1.1.2.2, GXG2 is a representative isolate of sub-genotype VI.2.1.1.2.1, and LaSota vaccine is representative of genotype II vaccines. The pink diagram represents the antigenicity analysis of the F protein, and the blue diagram represents the hydrophilicity analysis of the F protein. The rectangular areas highlight the antigenicity and hydrophilicity differences in the F proteins between different viruses. (**A**) 1. neutralizing antigen epitope region (72aa, 78aa, 79aa); 2. neutralizing antigen epitope region (157–171aa); neutralizing antigen epitope region (343aa); (**B**) 1. hydrophobic region 1, the signaling peptide region (1–25aa); 2. hydrophobic region 2, fusion induction region (117–142aa); 3. the seven-amino acid residue repeat region HRa (143–185aa); 4. the seven-amino acid residue repeat region HRc (268–289aa); 5. the seven-amino acid residue repeat region HRb (467–502aa); 6. hydrophobic region 3, transmembrane domain (500–521aa).

**Figure 3:**
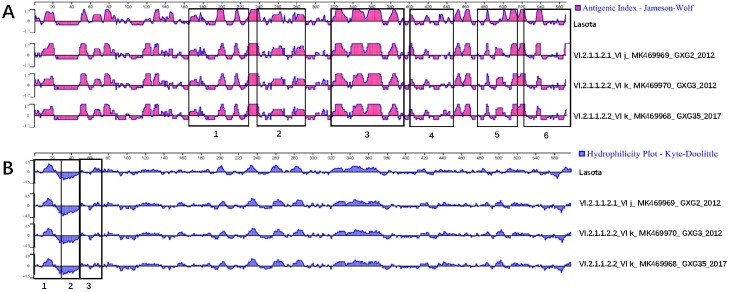
Comparison of the antigenicity and hydrophilicity of the HN of selected PPMV-1 and LaSota vaccine. Protean module of Laser gene was used to describe and analyze the antigenicity and hydrophilicity of hemagglutinin-neuraminidase (HN). GXG3 and GXG35 are representative isolates of sub-genotype VI.2.1.1.2.2, GXG2 is a representative isolate of sub-genotype VI.2.1.1.2.1, and LaSota vaccine is representative of genotype II vaccines. The pink diagram represents the antigenicity analysis of the HN, and the blue diagram represents the hydrophilicity analysis of the HN. The rectangular areas highlight the antigenicity and hydrophilicity differences in the HN between different viruses. (**A**) 1. HN helical folding structures: region 1 (175–228 aa); region 2 (237–288 aa); region 3 (316–396 aa); region 4 (401–443 aa); region 5 (472–515 aa); and region 6 (521–577 aa); (**B**) signal peptide region in cytoplasm (tail, 1–26 aa); 2. transmembrane region (rod-shaped, 27–48aa); 3. extracellular amino acids region (spherical head, 49–75aa).

Serum cross-inhibition confirmed that all pigeon-origin viruses studied here have antigenic identity between them, with *R* values between 0.85 and 1.00. Comparative analysis of the studied PPMV-1 with the LaSota vaccine showed antigenic relatedness. However, most of the *R* values were below 0.8, indicating the presence of some antigenic differences between LaSota and the studied PPMV-1 (Table 3). Additional antigenicity analysis using the Protean software predictions showed that F and HN protein sites were similar to those of the LaSota vaccine with overall close relatedness. However, the antigenicity analyses showed that some minor differences are present in the antigenic sites of the F and HN proteins of the two types of viruses (field PPMV-1 and vaccine) (Figure 2A and Figure 3A). The analyses also demonstrated that the compared PPMV-1 have different hydrophilicity profiles owing to amino acid substitutions in all three hydrophobic regions of the signal peptide region, transmembrane area, and fusion induction region of F and HN proteins. In addition, the repeated heptapeptide regions of F protein and signal peptide, spherical head, and helical folding regions of the HN also showed minor differences in the hydrophilicity prediction compared to the Lasota vaccine (Figure 2B and Figure 3B). The amino acid differences in F and HN proteins are summarized in Supplemental Tables S1, S2, and S3.

## 4. Discussion

In this study, we used deep sequencing to obtain the complete genomes of 18 pigeon-origin NDV isolated in China between 2012 and 2018. The isolates were characterized as members of genotype VI, which is typical for the antigenic variant of NDV referred to as PPMV-1. The isolated viruses were classified into two genotype VI sub-genotypes and showed further separation into several sub-groups. While these 18 viruses were isolated from diseased pigeons, biological evaluation of the viral pathogenicity showed that they are of low to no pathogenicity for chickens. Cross-reactivity and antigenicity analyses showed minor differences to the LaSota vaccine. The performed complete genome sequencing, epidemiological characterization, and biological pathogenicity evaluation provide useful information that will facilitate future surveillance, viral ecology studies, and disease control.

The isolated 18 PPMV-1 were characterized as low or avirulent to chickens despite possessing a virulent cleavage site. Commonly, the pathogenicity of NDV is evaluated by bioassays, estimating MDT and ICPI, and virulence is evaluated by analysis of the deduced amino acid motif at the F protein cleavage site [19,33]. For the vast majority of chicken-origin viruses, there is an agreement between virulent cleavage site motif (multiple basic amino acids between positions 113 and 116 and phenylalanine at position 117) and MDT (<60 h) and ICPI (above 0.7) values. However, some PPMV-1 are an exception. While all PPMV-1 have been shown to possess deduced amino acid motifs, suggestive of high virulence, most strains are mesogenic or lentogenic for chickens, as assessed through ICPI and MDT testing [34,35]. Nevertheless, the isolated viruses were causing clinical disease in pigeons. These findings suggest that the cleavage site is not the only factor influencing the pathogenicity of PPMV-1 in different species, and additional determinants impact the pathogenic phenotype of these viruses, as also reported by others [36]. For example, a recent study of virulent viruses of chicken- and pigeon-origin demonstrated differences in the infectivity and transmissibility of these viruses in chickens [37]. While both types of viruses infected chickens and were actively shed, the pigeon-origin virus did not cause mortality, consistent with previous studies [38]. Despite that many PPMV-1 are characterized as being of low or moderate pathogenicity for chickens, their potential to cause disease in poultry should not be underestimated [24,39]. Indeed, it has been reported that only several point mutations are sufficient to increase the pathogenicity of PPMV-1 in chickens [35,40]. Adaptation to chickens after serial passages with increased frequency of clinical signs, mortality, and the level of neuro-invasiveness has been demonstrated [41,42]. Pigeons are reported to also get infected with viruses from other NDV genotypes, including viruses that are pathogenic for chickens. In addition, not all viruses of genotype VI have been demonstrated to be PPMV-1. The current molecular tests cannot distinguish chicken-adapted NDV from PPMV-1 and, therefore, biological characterization of pathogenicity and risk analysis to the poultry industry is always warranted when an NDV is isolated from pigeons. It is unclear if the viruses studied here were of high pathogenicity for pigeons and the die offs were caused solely by them. A consideration can be given to the possibility that the identified PPMV-1 were of moderate pathogenicity for pigeons and the outbreaks were of multi-etiological nature.

The 18 viruses studied here belong to sub-genotypes VI.2.1.1.2.1 and VI.2.1.1.2.2, which are the predominant viruses affecting pigeons in China in the last decade. These viruses are genetically distant to the viruses of sub-genotypes VI.1 (7.8% and 8.4% nucleotide distance, respectively) and VI.2.2.2 (8.4% and 9.3%, respectively). The viruses of sub-genotypes VI.1 (former VIb) and VI.2.2.2 (former VIe) caused outbreaks in pigeons worldwide and in China during the 1990s and 2000s. However, there have been only a handful of isolates of these viruses during the last 10 years. In the late 2000s and the early 2010s, the viruses of sub-genotypes VI.1 and VI.2.2.2 were replaced by those of sub-genotypes VI.2.1.1.2.1 and VI.2.1.1.2.2 in China, and there are now hundreds of isolations of the latter [3,16,43]. With only 93.7% and 92.8% nucleotide identity, the viruses of sub-genotypes VI.2.1.1.2.1 and VI.2.1.1.2.2 are also genetically different from the viruses of sub-genotype VI.2.1.1.1 (former Via and VIn) causing outbreaks in pigeons in the United States.

The viruses of sub-genotypes VI.2.1.1.2.1 and VI.2.1.1.2.2 have been maintained in China for at least 15 years. The earliest identification of these viruses dates back to 2005. The viral reservoir in this case remains unknown. It is possible that the viruses have been maintained in pigeon farms or wild pigeon populations. Viruses of genotype VI have been previously reported to circulate in apparently healthy pigeons [12,44]. There have been reports of PPMV-1 being maintained in healthy captive [24,44] or wild pigeons [23].

Pigeon-derived viruses of sub-genotypes VI.2.1.1.2.1 and VI.2.1.1.2.2 have been identified in at least 19 Chinese provinces. Pigeons are not migratory birds and the mechanism of spread of these viruses at long distances between Chinese regions (and between countries) remains unknown. While there have been some speculations about the role of migratory birds in the spread of PPMV-1, it is doubtful that these birds are actively involved in the transmission of genotype VI viruses. There is no evidence of common infections of wild non-columbiform birds with PPMV-1 and such viruses are only occasionally isolated from migratory birds [3,45]. A viable hypothesis for the spread of PPMV-1 is the contact between Columbiform birds during competition flights (racing pigeons), exhibitions (show pigeons), live bird markets (meet pigeons), or intensive trade [24,46]. The active viral shedding from apparently healthy infected pigeons facilitates the unnoticed spread of the virus through these possible modes.

The performed phylogenetic analysis suggests that the viruses of sub-genotypes VI.2.1.1.2.1 and VI.2.1.1.2.2 have been independently and continuously evolving after the initial introduction to China. Both groups of viruses evolved from a common ancestor after initial introduction in the past or represent separate introductions of viruses that evolved elsewhere. The phylogenetic analysis shows that it is possible that both viral populations were introduced to China from Europe (Belgium or Italy, Figure 1, Supplemental Figures S1 and S2). However, such results should be interpreted with caution owing to sampling bias and limitations of the globally available genetic information for PPMV-1. Such molecular epidemiologic analyses rely on convenience, self-submitted genetic data, which may impact the phylogenetic inferences, and it is not unlikely that there is undetected diversity of PPMV-1 that can change the phylogeography inference. Moreover, as described above, often, infections with PPMV-1 do not cause clinical signs, and such cases may remain undetected and underreported. Most of the PPMV-1 sub-genotypes seem to be limited to certain world regions. Viruses of sub-genotypes VI.2.1.1.1 and VI.2.2.1 are found only in the United States, VI.2.2.2 are limited to China, and VI.2.1.2 are commonly identified in Africa with the exception of two viruses from South America. Similarly, viruses of sub-genotype XXI.2 are mostly found in Europe and those of sub-genotype XXI.1.2 are commonly isolated only in Pakistan. The viruses of sub-genotypes VI.1, VI.2.1.1.2.1, VI.2.1.1.2.2, and XXI.1.1 are an exception and are globally distributed. Additional historic and prospective virus and surveillance data will allow a more detailed phylogeographic analysis and inference of the epidemiological relationships of PPMV-1.

The complete genome phylogenetic analysis performed here was in agreement with the full fusion gene analysis (as per the most recent classification system). It is worth mentioning that, in many cases, complete genome phylogenetic reconstruction provides more reliable phylogenetic inferences [47], has better resolution, and allows resolving issues like low branch support and polytomies [3]. However, the lack of sufficient complete genome sequences in some NDV sub/genotypes (including PPMV-1 genotypes), at this time, prevents the development of a classification system that utilizes complete genomic data. With the increasing use of high-throughput sequencing technologies, the availability of complete genomes will also increase in the near future. Hopefully, the use of these enriched genomic databases and high performance computers will allow for the design of a complete genome classification system agreed on by a wide group of international experts.

The LaSota vaccine does not provide sufficient protection for pigeons from PPMV-1, as demonstrated by the multiple disease outbreaks in pigeon farms despite the wide use of this vaccine. Although minor, the antigenicity and hydrophilicity differences between the studied PPMV-1 and the LaSota vaccine identified here may contribute to this insufficient protection [48,49]. The use of only cross-HI assay and *in silico* assessment of the antigenicity and hydrophobicity of the isolates is a partial limitation of the present study. Further testing using virus neutralization assay and challenge studies is necessary to confirm the efficiency (or lack thereof) of the LaSota vaccine to protect against PPMV-1.

Up to date, there is no vaccine specifically developed to control Newcastle disease in pigeons in China. The sector is continuously growing and development of a vaccine that targets currently circulating strains is probably warranted. PPMV-1 are virulent viruses and the use of a live PPMV-1 vaccine is not a viable solution. However, other available live vaccines, similarly to LaSota, are chicken-adapted and will likely not be fully protective. Development of efficient antigenically matched inactivated vaccines with safe and strong adjuvants may facilitate the control of ND in pigeons.

## Figures and Tables

**Table 1:** Details of the pigeon paramyxoviruses 1 (PPMV-1) samples isolated in this study. HA, hemagglutination assay; MDT, mean death time; ICPI, intracerebral pathogenicity index; NGS, next-generation sequencing.

Isolates	GenBank Accession No.	Isolation Date	Flock Size	Clinical Signs and Gross Lesions	HA Titer	MDT	ICPI	NGS Raw Reads	Filtered Reads	PPMV-1 Reads	Genotype
GXG1	MK749297	Nov 2012	800	neurological symptoms, diarrhea	log_2_8	76 h	0.00	15,033,538	14,121,738	7,788,836	VI.2.1.1.2.2 (VIk)
GXG2	MK469969	Mar 2012	4000	neurological symptoms, hemorrhages in glandular stomach	log_2_8	94 h	0.00	14,119,609	13,203,568	1,630,974	VI.2.1.1.2.1 (VIj)
GXG3	MK469970	May 2012	1000	hemorrhages in glandular stomach and peritonitis	log_2_9	72 h	0.25	13,767,808	12,781,443	1,755,997	VI.2.1.1.2.2 (VIk)
GXG6	MK469971	May 2013	2500	diarrhea, hemorrhages, and necrosis in the spleen	log_2_7	92 h	0.00	14,546,579	13,527,020	2,989,862	VI.2.1.1.2.2 (Vik)
GXG7	MK469972	Feb 2014	3600	neurological symptoms, diarrhea	log_2_10	81 h	0.25	15,209,534	14,368,697	4,024,270	VI.2.1.1.2.1 (VIj)
GXG13	MK469973	Mar 2015	2000	neurological symptoms	log_2_7	62 h	0.17	14,891,909	13,844,914	3,051,814	VI.2.1.1.2.2 (VIk)
GXG16	MK469974	May 2015	2000	neurological symptoms, diarrhea	log_2_4	69 h	0.63	13,082,370	12,098,553	1,630,974	VI.2.1.1.2.1 (VIj)
GXG20	MK469964	Jun 2015	1000	neurological symptoms	log_2_7	78 h	0.00	10,876,496	9,871,185	2,172,462	VI.2.1.1.2.2 (VIk)
GXG22	MK749298	Jul 2015	400	hemorrhages in glandular stomach, neurologic symptoms	log_2_5	80 h	0.00	12,076,535	11,249,343	1,306,032	VI.2.1.1.2.2 (VIk)
GXG24	MK749299	Jul 2015	1200	diarrhea, hemorrhages, and necrosis in the spleen	log_2_3	105 h	0.00	13,863,796	12,818,138	1,489,872	VI.2.1.1.2.2 (VIk)
GXG25	MK749300	Aug 2015	1200	hemorrhages in glandular stomach, neurologic symptoms	log_2_3	96 h	0.00	14,886,898	13,994,957	4,915,176	VI.2.1.1.2.2 (VIk)
GXG6	MK469966	Sep 2015	2200	neurological symptoms, diarrhea	log_2_3	114 h	0.00	10,669,774	10,033,373	2,887,488	VI.2.1.1.2.1 (VIj)
GXG28	MK749301	Jan 2016	1000	diarrhea, hemorrhages in glandular stomach	log_2_4	90 h	0.00	17,058,483	15,953,502	7,449,482	VI.2.1.1.2.2 (VIk)
GXG29	MK469965	Feb 2016	4000	neurological symptoms	log_2_5	70 h	0.00	14,457,740	13,485,935	7,797,452	VI.2.1.1.2.2 (VIk)
GXG31	MK749302	Mar 2016	4000	neurological symptoms	log_2_3	93 h	0.00	13,384,970	12,544,326	2,102,690	VI.2.1.1.2.2 (VIk)
GXG33	MK469967	Apr 2016	1200	neurological symptoms	log_2_6	69 h	0.45	9,915,567	9,426,738	1,350,934	VI.2.1.1.2.2 (VIk)
GXG35	MK469968	Jun 2017	2600	hemorrhages in glandular and stomach	log_2_3	98 h	0.00	12,150,471	10,993,904	1,713,840	VI.2.1.1.2.2 (VIk)
GXG44	MN477454	Dec 2018	3000	neurologic symptoms, diarrhea	log_2_4	96 h	0.00	15,825,591	14,138,234	4,824,912	VI.2.1.1.2.2 (VIj)

**Table 2:** Estimates of evolutionary distance between different sub-genotypes of class II genotype VI Newcastle disease viruses.

	Number of Base Substitutions per Site ^a^					
	VI.1	VI.2.2.1	VI.2.2.2	VI.2.1.1.1	VI.2.1.2	VI.2.1.1.2.1
VI.1						
VI.2.2.1	6.5					
VI.2.2.2	7.2	6.2				
VI.2.1.1.1	8.5	8.3	9.0			
VI.2.1.2	8.2	8.9	9.3	7.9		
VI.2.1.1.2.1	7.8	7.6	8.4	6.3	7.8	
VI.2.1.1.2.2	8.4	8.5	9.3	7.2	8.9	5.3

^a^ The number of base substitutions per site from averaging over all sequence pairs between groups is shown. Analyses were conducted using the maximum composite likelihood model [29]. The rate variation among sites was modeled with a gamma distribution (shape parameter = 1). The analysis involved 282 nucleotide sequences. Codon positions included were 1st+2nd+3rd+noncoding. There were a total of 1659 positions in the final dataset. Evolutionary analyses were conducted in MEGA6 [28].

**Table 3:** Serum cross-reactivity *R* value results by hemagglutination inhibition assay.

Ab/Ag	Lasota	GXG2	GXG7	GXG16	GXG6/2015	GXG1	GXG3	GXG6/2013	GXG13	GXG24
**Lasota**										
**GXG2**	0.75									
**GXG7**	0.77	0.91								
**GXG16**	0.79	0.90	0.93							
**GXG6/2015**	0.75	0.94	0.89	0.95						
**GXG1**	0.66	0.92	0.92	0.85	0.88					
**GXG3**	0.81	0.98	1.00	0.97	0.94	0.98				
**GXG6/2013**	0.79	0.90	0.91	0.88	0.85	0.85	0.94			
**GXG13**	0.71	0.91	0.89	0.91	0.85	0.89	1.00	0.92		
**GXG24**	0.78	0.90	0.85	0.90	0.85	0.85	0.92	0.88	0.87	
**GXG25**	0.68	0.93	0.89	0.88	0.87	0.92	0.97	0.91	0.93	1.00

## Supplementary Materials

The following are available online at https://www.mdpi.com/1999-4915/12/4/366/s1, Figure S1: Phylogenetic analysis based on the full-length nucleotide sequence of the fusion gene of all available isolates representing Newcastle disease virus class II. The evolutionary history was inferred by using the maximum likelihood method based on the general time reversible model with 1000 bootstrap replicates. The tree with the highest log likelihood (–113,734.64) is shown. A discrete gamma distribution was used to model evolutionary rate differences among sites. The analysis involved 1787 nucleotide sequences with a total of 1651 positions in the final dataset. The viruses isolated in this study are designated with yellow highlight. The Roman numerals presented in the taxa names in the phylogenetic tree represent the respective sub/genotype for each isolate, followed by the GenBank identification number, host name, country of isolation, strain designation, and year of isolation (if available). Figure S2: Phylogenetic analysis based on the full-length complete genome coding sequences of available isolates representing Newcastle disease virus class II genotype VI. The evolutionary history was inferred by using the maximum likelihood method based on the general time reversible model with 1000 bootstrap replicates. The tree with the highest log likelihood (–91,564.38) is shown. A discrete gamma distribution was used to model evolutionary rate differences among sites. The analysis involved 108 nucleotide sequences with a total of 13,595 positions in the final dataset. The viruses isolated in this study are designated with yellow highlight. The Roman numerals presented in the taxa names in the phylogenetic tree represent the respective sub/genotype for each isolate, followed by the GenBank identification number, host name, country of isolation, strain designation, and year of isolation (if available). Table S1: Amino acid substitutions in the functional domains of the fusion protein. Table S2: Amino acids substitutions at the trans-membrane domain and the neutralizing epitopes of HN. Table S3: Sub-genotype specific amino acid positions in F and HN proteins.
