# Supplementary material for: Complete Genome Sequencing, Molecular Epidemiological, and Pathogenicity Analysis of Pigeon Paramyxoviruses Type 1 Isolated in Guangxi, China during 2012–2018
Source: Viruses. 2020 Mar 26;12(4):366. doi: 10.3390/v12040366 (PMC7232316; doi:10.3390/v12040366)
Supplement: Supplementary file 1 [file viruses-12-00366-s001.zip › Supplemental Table S1.docx]

Supplemental Table 1. Amino acid substitutions in the functional domains of the fusion protein.

| **Lasota/Clone30/B1** | **Signal peptide**  **1-25** | **Cleavage site**  **112–117** | **Fusion peptide**  **117-142** | **HRa 143-185** | **HRc268-299** | **HRb 471-500** | **Trans-membrane**  **domain 501–521** | **N-linked glycosylaiton sites** | **Cysteine residue**  **sites** |
| --- | --- | --- | --- | --- | --- | --- | --- | --- | --- |
|  | **MGSKPHTRNPA**  **PMMLITRITLVLS** | **GRQGR↓L** | **FIGAIIGSVALGVATAAQITAAAALI** | **QANQNAANILRLKESIAATNEAVHEVTDGLSQLAVAVGKMQQF** | **LITGNPILYDSQTQLLGIQVNLPSVGNLNNMR** | **NNSISNALDKLAESNSKLDKVNVKLTSTSA** | **LITYIVLTVISLVFGILSLVL** | **85,191,366,**  **447,471,541** | **27,76,199,**  **347,401,514,** |
| Pi/Belgium/98-248/1998 | H6Y/N9I/M13L | RRQKR↓F | A132S | - | - | - | I516A | - | - |
| Pi/GX/1015/13 | N9T/M13P | RRQKR↓F | A132S | V168I, | - | K480R | I502V/I516T | - | - |
| GXG2 | N9T/M13P | RRQKR↓F | A132S | V168I, | - | N471K, | I502V/I516T | N471K | - |
| GXG7 | N9T/M13P | RRQKR↓F | A132S | V168I, | - | - | I502V/I516T | - | - |
| GXG16 | N9T/M13P | RRQKR↓F | A132S | V168I, | - | - | I502V/I516T | - | - |
| GXG6/2015 | K4E/N9T/M13P | RRQKR↓F | A132S | V168I, | - | - | I510M/I516T | - | C27R |
| GXG44 | N9T/M13P | RRQKR↓F | A132S | V168I, | - | - | I510M/I516T |  |  |
| Pi/Belgium/11-07574/2011 | H6S/7TI/N9I/M13L/M14T | RRQKR↓F | A132S | V179I | - | K480R | V506I/ I516A | - | - |
| GXG1 | G2D/7TI//N9I/ M13P/ M14T | RRQKR↓F | A132S | V179I | - | K480R | V506I/ | - | - |
| GXG3 | G2D/7TI/N9I/M13P/M14T | RRQKR↓F | A132S | V179I | - | K480R | V506I/ I516A | - | - |
| GXG6/2013 | G2D/7TI/N9T/M13P/M14T/V22L | RRQKR↓F | A132S | V179I | - | K480R/N485D | V506I/ I516A | - | - |
| GXG13 | 7TI/N9I/M13L/M14T | RRQKR↓F | A132S | V179I | - | K480R | V506I/ I516A | - | - |
| GXG20 | 7TI/ N9I/M13L/M14T | RRQKR↓F | A132S | V179I | - | K480R | V506I/T508N/I516A | - | - |
| GXG22 | 7TI//M13L/ M14T | RRQKR↓F | A132S | V179I | - | K480R | V506I/ T508N/ I516A | - | - |
| GXG24 | 7TI//M13L/ M14T | RRQKR↓F | A132S | V179I | - | K480R | V506I/ I516A | - | - |
| GXG25 | 7TI/ M13L/ M14T | RRQKR↓F | A132S | V179I | - | K480R | V506I/ I516A | - | - |
| GXG28 | 7TI/ M13L/ M14T | RRQKR↓F | A132S | V179I | - | K480R | V506I/ I516A | - | - |
| GXG29 | G2D/7TI/N9I/M13P/M14T | RRQKR↓F | A132S | V179I | - | N476S/S486N | V506I/I516A | - | - |
| GXG31 | G2D/7TI//N9I/ M13P/ M14T | RRQKR↓F | A132S | V179I | - | K480R/S486N | V506I/ I516A | - | - |
| GXG33 | G2D/7TI/N9I/M13P/M14T | RRQKR↓F | A132S | V179I | - | K480R | V506I/ I516A | - | - |
| GXG35 | 7TI/ N9I/M13L/M14T/R18Q | RRQKR↓F | A132S | V179I | - | K480R | V506I/T508N/I516A | - | - |
| Chicken/GX11/2003 | H6S/N9I/M13L/T20M/V22I | RRQKR↓F | I121V | A176S | N272Y | D479E/ K480R/S486T/D489E | I509V/V513I | - | - |
| Dove/GX15/2005 | S3F/N9I/M13L/T20M/V22I/L24M | RRQKR↓F | I121V | N145K/D170N/A176S, | N272Y/Q279H | K480R/D489E/V491I/N492D/T496A | I509V/V513I/ I516A /S518I | - | - |
