# Supplementary material for: Complete Genome Sequencing, Molecular Epidemiological, and Pathogenicity Analysis of Pigeon Paramyxoviruses Type 1 Isolated in Guangxi, China during 2012–2018
Source: Viruses. 2020 Mar 26;12(4):366. doi: 10.3390/v12040366 (PMC7232316; doi:10.3390/v12040366)
Supplement: Supplementary file 1 [file viruses-12-00366-s001.zip › Supplemental Table S2.docx]

Supplemental Table 2. Amino acids substitutions at the trans-membrane domain and the neutralizing epitopes of HN.

| **Lasota /Clone30/ B1** | **Transmembrane domain** | **Neutralizing epitopes of HN protein** | | | | | | | | | |
| --- | --- | --- | --- | --- | --- | --- | --- | --- | --- | --- | --- |
|  | **27–48**  **IAVLLLTVVTL**  **AISVAALLYSM** | **193-201**  **LSGCRDHSH** | **263N** | **287D** | **321K** | **332-333**  **GK** | **345-353**  **PDEQDYQIR** | **401-443**  **EGRILTVGTSHFLYQRGSSYFS**  **PALLYPM TVSNKTATLHSPYT** | **494G** | **513-521**  **RITRVSSSS** | **569D** |
| Pi/Belgium/98-248/1998 | T33I/L45V | R197I | K | - |  | - | E347G/D349E | I404V/S432Y | D | - | - |
| Pi/GX/1015/13 | T33I/V41A/ L45V/M48 T | R197I | K | - | - | - | E347G/D349E /I352V | I404V/S432Y/T443M | D | I514V | G |
| GXG2 | L45V/T33I/Y46C | R197I | K | - | - | - | E347G/D349E /I352V | I404V/S432Y/S440N/T443M | D | I514V | G |
| GXG7 | L45V/T33I/A42S | R197I | K | - | - | - | E347G/D349E /I352V | I404V/S432Y/ S440N/T443M | D | I514V | G |
| GXG16 | T33I/L45V | R197I | K | - | - | - | E347G/D349E /I352V | I404V/S432Y/ S440N/T443M | D | I514V | G |
| GXG6/2015 | T33I/L45V | R197I | K | - | R | - | E347G/D349E /I352V | I404V/S432Y/ S440K/T443M | D | I514V | G |
| GXG44 | T33I/L45V | R197I | K | - | - | - | E347G/D349E /I352V | I404V/S432Y/ S440K/T443M | D | I514V | G |
| Pi/Belgium/11-07574/2011 | V29I/L45V | R197I | K | - | - | - | E347G/D349E- | I404V/S432H/S440N | D | I514V | E |
| GXG1 | L32F/L45I | R197I | K |  |  |  | E347G/D349E/ | I404V/S432H/S440N | D | I514V | E |
| GXG3 | L32F/L45I | R197I | K | - | - | - | E347G/D349E - | I404V/S432H/S440N | D | I514V | E |
| GXG6/2013 | L32F/L45I, | R197I | K | - | - | - | E347G/D349E | I404V/S432H/S440N | D | I514V | E |
| GXG13 | L45V | R197I | K | - | - | - | E347G/D349E | I404V/S432H/S440N | D | I514V | E |
| GXG20 | L45V | R197I | K | - | - | - | E347G/D349E | I404V/S432H/S440N | D | I514V | E |
| GXG22 | I39F/ L45V | R197I | K |  |  |  | E347G/D349E/ | I404V/S432H/S440N | D | I514V | E |
| GXG24 | L45V | R197I | K |  |  |  | E347G/D349E/ | I404V/S432H/S440N | D | I514V | E |
| GXG25 | L45V | R197V | K |  |  |  | E347G/D349E/ | I404V/S432H/S440N | D | I514V | E |
| GXG28 | L45V | R197I | K |  |  |  | E347G/D349E/ | I404V/S432H/S440N | D | I514V | E |
| GXG29 | V35A/ L45V/M48T | R197A | K | - | - | - | E347G/D349E - | I404V/S432H/S440N | N | I514V | E |
| GXG31 | V35A/ L45V/M48T | R197A | K |  |  |  | E347G/D349E/ | I404V/S432H/S440N | N | I514V | E |
| GXG33 | L45V | R197I | K | - | - | - | E347G/D349E - | I404V/S432H/S440N | D | I514V | E |
| GXG35 | L45V | R197I | K | - | - | - | E347G/D349E - | I404V/S432H/H439R/S440N | D | I514V | K |
| Chicken/GX11/2003 | T33M/V35M/V45A/M48T | - | K | - | - | - | Q348K | S432N | D | I514V | G |
