# Supplementary material for: Complete Genome Sequencing, Molecular Epidemiological, and Pathogenicity Analysis of Pigeon Paramyxoviruses Type 1 Isolated in Guangxi, China during 2012–2018
Source: Viruses. 2020 Mar 26;12(4):366. doi: 10.3390/v12040366 (PMC7232316; doi:10.3390/v12040366)
Supplement: Supplementary file 1 [file viruses-12-00366-s001.zip › Supplemental Table S3.docx]

Supplemental Table 3. Sub-genotype specific amino acid positions in F and HN proteins.

| **Strain / Site** | **Fusion Protein** | | | | | | | | | | | | | **Hemagglutinin-Neuraminidase Protein** | | | | | | | | | | | | | | | | | | | | |
| --- | --- | --- | --- | --- | --- | --- | --- | --- | --- | --- | --- | --- | --- | --- | --- | --- | --- | --- | --- | --- | --- | --- | --- | --- | --- | --- | --- | --- | --- | --- | --- | --- | --- | --- |
|  | **7** | **9** | **13** | **14** | **28** | **168** | **179** | **203** | **432** | **480** | **502** | **506** | **516** | **3** | **5** | **7** | **33** | **58** | **59** | **66** | **74** | **84** | **127** | **145** | **147** | **254** | **266** | **309** | **340** | **352** | **432** | **443** | **497** | **569** |
| Lasota /Clone30/ B1 | T | N | M | M | P | V | V | A | I | K | I | V | I | R | V | Q | T | G | I | A | L | I | I | A | D | V | V | D | Y | I | S | T | A | D |
| Pi/Belgium/98-248/1998 | - | I | L | - | L | - | - | T | V | R | - | - | A | H | - | K | I | - | - | - | - | V | V | I | G | - | T | N | - | - | Y | - | - | - |
| Pi/GX/1015/13 | - | T | P | - | - | I | - | T | V | - | V | - | T | H | I | K | I | - | - | - | - | V | V | V | S | I | T | N | H | V | Y | M | - | G |
| GXG2 | - | T | P | - | - | I | - | T | V | - | V | - | T | H | I | K | I | - | - | - | - | V | V | V | S | I | T | N | H | V | Y | M | - | G |
| GXG7 | - | T | P | - | - | I | - | T | V | - | V | - | T | H | I | K | I | - | - | - | - | V | V | V | S | I | T | N | H | V | Y | M | - | G |
| GXG16 | - | T | P | - | - | I | - | T | V | - | V | - | T | H | I | K | I | - | - | - | - | V | V | V | S | I | T | N | H | V | Y | M | - | G |
| GXG6/2015 | - | T | P | - | L | I | - | T | V | - | - | - | T | - | I | K | I | - | - | - | - | V | V | V | S | - | T | N | H | V | Y | M | - | G |
| GXG44 | - | T | P | - |  | I | - | T | V | - | V | - | T | H | I | K | I | - | - | - | - | V | V | V | S | I | T | N | H | V | Y | M | - | G |
| Chicken/GX11/2003 | - | I | L | - | L | - | - | T | - | R | - | - | A | - | - | R | M | - | - | T | - | - | V | I | D | - | A | - | H | - | N | - | - | G |
| Pi/Belgium/11-07574/2011 | I | I | L | T | S | - | I | S | - | R | V | I | A | H | - | K | - | - | V | V | I | - | - | I | G | - | T | - | - | - | H | - | - | E |
| GXG1 | I | I | P | T | S | - | I | S | - | R | - | I | A | - | - | K | - | S | V | V | I | - | - | I | G | - | T | - | - | - | H | - | T | E |
| GXG3 | I | I | P | T | S | - | I | S | - | R | - | I | A | - | - | K | - | S | V | V | I | - | - | I | G | - | A | - | - | - | H | - | T | E |
| GXG6/2013 | I | T | P | T | S | - | I | S | - | R | - | I | A | - | - | K | - | S | V | V | I | - | - | I | G | - | A | - | - | - | H | - | T | E |
| GXG13 | I | I | L | T | S | - | I | S | - | R | - | I | A | - | - | K | - | S | V | V | I | - | - | I | G | - | T | - | - | - | H | - | T | E |
| GXG20 | I | I | L | T | S | - | I | S | - | R | - | I | A | - | - | K | - | S | V | V | I | - | - | I | G | - | T | - | - | - | H | - | T | E |
| GXG22 | I | I | L | T | S | - | I | S | - | R | - | I | A | - | - | K | - | S | V | V | I | - | - | I | G | - | T | - | - | - | H | - | T | E |
| GXG24 | I | I | L | T | S | - | I | S | - | R | - | I | A | - | - | K | - | S | V | V | I | - | - | I | G | - | T | - | - | - | H | - | T | E |
| GXG25 | I | I | L | T | S | - | I | S | - | R | - | I | A | - | - | K | - | S | V | V | I | - | - | I | G | - | T | - | - | - | H | - | T | E |
| GXG28 | I | I | L | T | S | - | I | S | - | R | - | I | A | - | - | K | - | S | V | V | I | - | - | I | G | - | T | - | - | - | H | - | T | E |
| GXG29 | I | I | P | T | S | - | I | S | - | R | - | I | A | - | - | K | - | - | V | V | I | - | - | I | G | - | T | - | - | - | H | - | T | E |
| GXG31 | I | I | P | T | S | - | I | S | - | R | - | I | A | - | - | K | - | S | V | V | I | - | - | I | G | - | T | - | - | - | H | - | T | E |
| GXG33 | I | I | P | T | S | - | I | S | - | R | - | I | A | - | - | K | - | - | V | V | I | - | - | I | G | - | T | - | - | - | H | - | - | E |
| GXG35 | I | I | L | T | S | - | I | S | - | R | - | I | A | - | - | K | - | S | V | V | I | - | V | I | G | - | T | - | - | - | H | - | T | K |
