# Supplementary material for: Complete Genome Sequencing, Molecular Epidemiological, and Pathogenicity Analysis of Pigeon Paramyxoviruses Type 1 Isolated in Guangxi, China during 2012–2018
Source: Viruses. 2020 Mar 26;12(4):366. doi: 10.3390/v12040366 (PMC7232316; doi:10.3390/v12040366)

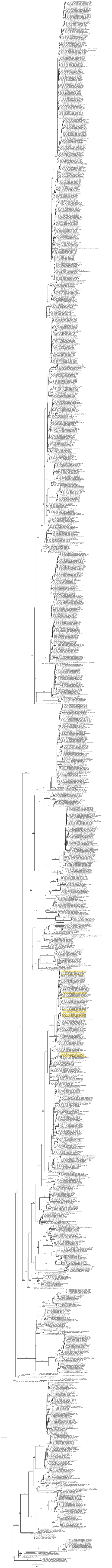

Supplement: Supplementary file 1 [file Supplemental Figure S1.pdf › Supplemental Figure S1.pdf]
